# Layers: A molecular surface peeling algorithm and its applications to analyze protein structures

**DOI:** 10.1038/srep16141

**Published:** 2015-11-10

**Authors:** Naga Bhushana Rao Karampudi, Ranjit Prasad Bahadur

**Affiliations:** 1 Advanced Technology Development Center, Indian Institute of Technology Kharagpur, Kharagpur-721302, West Bengal, India; 2 Computational Structural Biology Lab, Department of Biotechnology, Indian Institute of Technology Kharagpur, Kharagpur-721302, West Bengal, India

## Abstract

We present an algorithm ‘Layers’ to peel the atoms of proteins as layers. Using Layers we show an efficient way to transform protein structures into 2D pattern, named residue transition pattern (RTP), which is independent of molecular orientations. RTP explains the folding patterns of proteins and hence identification of similarity between proteins is simple and reliable using RTP than with the standard sequence or structure based methods. Moreover, Layers generates a fine-tunable coarse model for the molecular surface by using non-random sampling. The coarse model can be used for shape comparison, protein recognition and ligand design. Additionally, Layers can be used to develop biased initial configuration of molecules for protein folding simulations. We have developed a random forest classifier to predict the RTP of a given polypeptide sequence. Layers is a standalone application; however, it can be merged with other applications to reduce the computational load when working with large datasets of protein structures. Layers is available freely at http://www.csb.iitkgp.ernet.in/applications/mol_layers/main.

Every algorithm that can help to explain biology with a new perspective is critical for the evolution of biological science. Digitization of biological data have stirred up and revolutionized the speed of exploring biology, provided suitable algorithms exist. Structural data in Protein Data Bank (PDB)^1^ is increasing exponentially and the available algorithms are not sufficient enough to explain many biological functions from these data. Comprehensive knowledge of one protein can be extended to explain the properties of similar proteins^2^. However, similarity can be described in terms of surface properties, molecular shape, molecular functions, structural architecture and folding pattern. Comparative studies on similar protein structures can reveal the key to functional diversity^3^,^4^. Similarity between molecules is a crucial feature that is exploited in molecular modeling, molecular docking, mutagenesis studies and drug designing. Identifying similar proteins starts with amino acid sequence or structure comparison methods. However, these methods are limited by failing to account for evolutionary distance^5^ or sometimes computationally expensive^6^,^7^,^8^,^9^. Pre-classifying the protein database^7^,^10^,^11^ is fuzzy as proteins may not exist in clearly classifiable clusters^4^,^12^ and feature based dynamic retrieval of similar molecules involves many to many comparisons making it impractical for working with large datasets^7^,^8^,^9^. Reducing or transforming the structural information into patterns allows large scale comparison faster. Shape signature is one such transformation, which shows their potential in molecular recognition and drug designing^13^,^14^.

Protein pairs may not qualify as similar if molecules are compared as a whole, but local regions could make them similar by comparing active sites or binding sites using local similarity search^15^,^16^. Moreover, search space can be reduced by using patterns or coarse-grain model to represent a molecule. Structures, being better conserved than sequence^17^, make the comparison between proteins more reliable than sequences for identifying molecular similarity; yet structure based methods often fails to identify similar structures^18^,^19^.

In a linear polypeptide, residues at distant positions interact and form a nucleus, which grows into a fully folded structure by wrapping the surrounding residues^20^. Viewing molecules as layers of atoms, it can be hypothesized that the surface layer is stabilized by the inner layers. In this study, we have developed an algorithm named Layers that peels atoms of molecules as layers from periphery to center. Layers identifies residue transition pattern (RTP), which may be used for the comparison of folding pattern between two molecules. Besides, it also extracts molecular surfaces and protruding atoms at custom fineness by non-random sampling, which can be used for shape estimation and molecular surface comparison for protein recognition and ligand design. We also present a comparison of surface layers extracted using Layers and Solvent Accessible Surface Area (SASA) method^21^.

## Results

### Peeling of molecular surface using Layers

**Figure 1:**
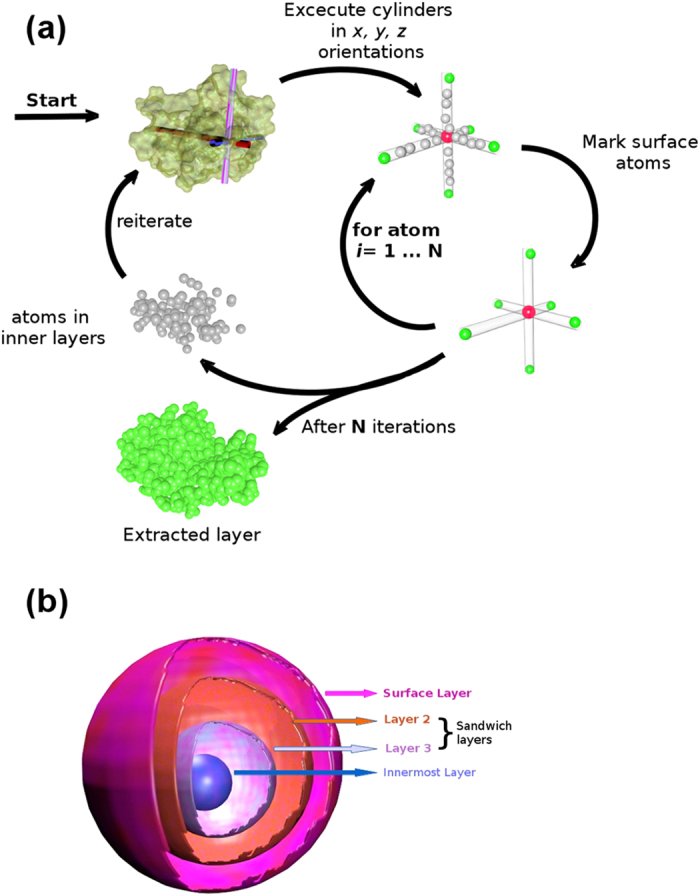
Description of Layers algorithm. (**a**) Schematic representation of ‘Layers’ algorithm. In a given macromolecular structure, Layers define three cylinders for each atom (coloured red) along the x-, y- and z- axes. Each cylinder engulfs atoms in them. The terminal atoms (coloured green) in each cylinder are assigned to a layer. This process is iterated for all atoms (N) present in the structure to define a layer. Atoms that are not selected in the present layer (coloured gray) are subject to be iterated for next layer. These iterations continue as long as all the atoms are labeled in layers. (**b**) Hierarchical arrangement of molecular layers. First layer peeled for a molecule is the surface layer, followed by the sandwich layers (layers 2, 3 and so on), and the innermost layer (IM).

Layers peels molecular surface by defining three cylinders passing through a given atom, each oriented along *x, y* and *z* axis (Fig. 1a). For a given atom, it identifies other atoms whose center of mass is engulfed by these cylinders having a default radius (*Sr*) of 1.52 Å, equivalent to the smallest atom in the protein crystals^21^. Length of the cylinder dynamically extends or shrinks to accommodate the atoms. Periphery atoms in these cylinders are collected as a layer. Atoms left uncollected are assigned into inner layers, which can be extracted further as layers by iterations. This continues till no more atoms are left unassigned. First layer extracted for a molecule is termed as the surface layer, final layer is termed as the innermost (IM) layer and the layers in between are termed as the sandwich layers (Fig. 1b). Radius of the cylinder, *Sr* is a crucial parameter, which can be used to sample the surface non-randomly. Increased *Sr* reduces the fineness of the peeled surface, yet useful to capture the shape of the molecule.

**Figure 2:**
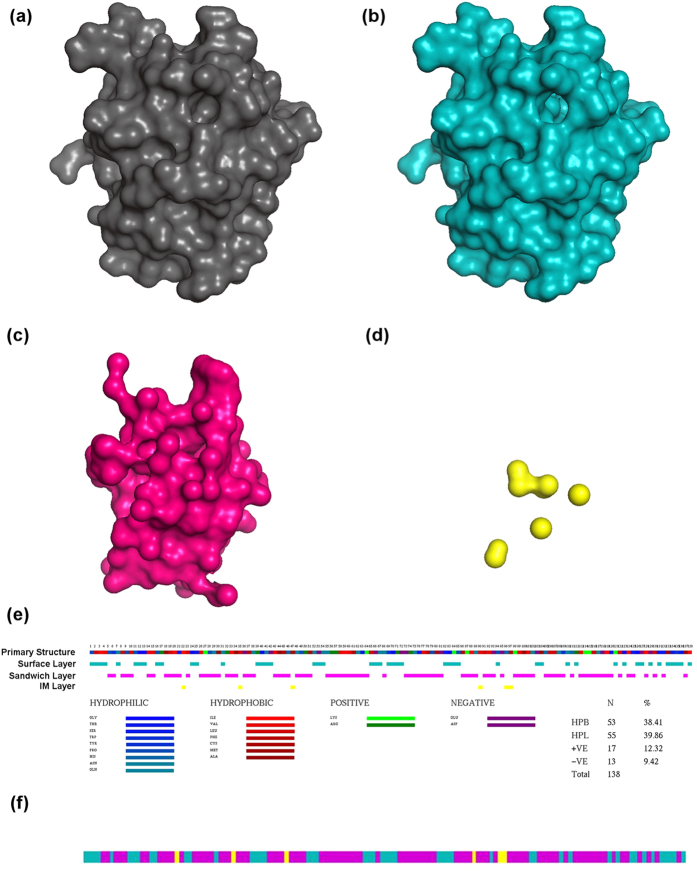
Representation of molecular surface and the layers extracted from the carbohydrate recognition domain of human galectin-3 (PDB id: 3ZSJ) and its corresponding RTP. (**a**) Molecular surface of the native structure. (**b**) Surface layer or the first peeled layer of the molecule. Inside of this structure is completely hollow. (**c**) Sandwich layer or second layer. (**d**) IM layer or the last possible layer that can be peeled for this structure. (**e**) Primary structure of the protein associated with the RTP obtained from peeled layers. Primary sequence is color coded according to their classification and layers as per the color code provided. Colour scheme is according to the hydrophobic scale. Hydrophilic (HPL): Gly, Thr, Ser, Trp, Tyr, Pro, His, Asn, Gln; Hydrophobic (HPB): Ile, Val, Leu, Phe, Cys, Met, Ala; Positive: Lys, Arg; Negative: Glu, Asp. The total number (N) and the percentage composition of residues are shown in the bottom right panel. (**f**) RTP transformed into 1D (IM layer in yellow, sandwich layers in pink and surface layer in cyan colors).

Figure 2 depicts the peeled layers of carbohydrate recognition domain (CRD) of human galectin-3 (Fig. 2a–d). Layers peeled three layers for this protein. The native structure and the peeled surface layer are shown in Fig. 2a,b, respectively. The peeled surface layer is completely hollow inside. Figure 2c is the sandwich layer as it happens to appear between the surface and the IM layers. Figure 2d is the IM layer, the final layer that can be peeled for this structure. Now to transform the atoms layer information into residue layers we traverse from the IM layer to the surface layer. First occurrence of a particular residue in a layer is assigned to that layer in which it appears, while ignoring its presence in the next layers or layers on top of it. The rationale behind this assignment is that, when a protein folds the preordained position of residues in the tertiary structure is established and stabilized by the atoms that interacts with the growing nucleus^20^. Hence, these interacting and stabilizing atoms get buried in the inner sides of the structure and appears first when we traverse from inside to the periphery of the structure. Figure 2e shows a superimposed image of a primary sequence and the residue transition pattern (RTP) obtained using Layers. By traversing from IM layer to surface layer, the information of atoms in layers is assigned to residues. Now the obtained RTP shows that, residues 1 to 4 are in the surface layer, residues 5 and 6 are in the second layer and so on. Residues 22, 35, 47, 90, 96 and 97 are found in the IM layer, which constitutes the center of the protein. The residues in the IM layer have two distinct boundaries in sequence space separated by 43 residues; one at the N-terminal and the other at the C-terminal. IM layer is wrapped up by the residues constituting the next layer, which is discontinuous as the intermittent parts of the polypeptide weaves in and out of the molecule forming the surface and the sandwich layers. RTP facilitates residue wise comparisons for its properties and corresponding layer along with its position in the primary sequence and in the tertiary structure (Fig. 2e). Protein folding pattern as such refers to the events involved in transforming the linear polypeptide sequence into a folded structure. RTP identifies the residue transition in folded structures, hence it can be considered as final snap shot of folding pattern of a molecule. RTP can also help to identify residues that are safe to mutate without affecting the structural stability. Mutating residues on the surface layer may not disturb the stability and the folding of the molecule^22^, whereas, residues in the sandwich layers may be mutated to understand the folding property of the molecule. Residues in the IM layer represent the core of the protein and are very crucial for the stability of the tertiary structure; hence, these residues should be carefully mutated. This analysis can contribute to the experimental design of mutagenesis studies. RTP can be transformed into single line multi-colored pattern (Fig. 2f) emphasizing layers information to facilitate large scale comparison of folding patterns of molecules, which is discussed in the later sections.

Barnase is one of the model proteins which has been extensively studied to understand protein folding^23^. The X-ray structure of barnase (PDB id: 1A2P) contains 110 amino acid residues of which 48% hydrophilic, 28% hydrophobic, 13% positively charged and 11% negatively charged, while Cys and Met are absent. Propensity of residues in the surface and in the IM layers is shown in Supplementary Table S1. Layers extracted two layers for barnase: the IM layer and the surface layer. The resulting RTP is shown in Supplementary Fig. 1. RTP shows continuous and frequent transition of residues between the IM and the surface layers. We find 40% residues of the barnase are in the surface layer while 60% are in the IM layer. Because of the inherent dominance of the hydrophilic residues in the polypeptide sequence, IM layer is also dominated by the hydrophilic residues followed by the hydrophobic, positively and negatively charged residues. However, the unbiased view of residue types occupying layers is shown with propensity values in Supplementary Table S1. Negatively charged residues show high tendency towards the surface layer, while hydrophobic residues show high tendency towards the IM layer. Maximum number of transitions between the IM and the surface layers are found between 33^rd^ and 67^th^ positions along the sequence, which accompany 80% of glycine present in barnase. Glycine may facilitate the transition of residues between the layers. The longest and continuous stretches of residues in IM layer (≥8 residues) appears between 69–76 and 84–92 residue positions, which are accompanied with ≥3 salt bridges. The second longest continuous stretches of IM layers (≥4 residues) between 24–28 and 50–53 residue positions are accompanied by one salt bridge. IM layer with a continuous stretch of three residues are accompanied by a salt bridge with only exception at the C-terminal. The salt bridge found at the C-terminal is not close to the IM layer. All the beta sheets are found in the IM layer, while the helices traverse between the IM and the surface layers.

The existence of intermediate in the folding of barnase is still debatable^24^,^25^. Since RTP is generated from the final snap shot of a folded structure, it may not help to understand the presence or absence of intermediates during folding event. However, RTP clearly signifies that the residues are continuously weaving in and out between the IM and the surface layers without showing any clear clustering of them in the IM layer.

### Comparison of protein folding patterns using RTP

Sometimes evolution converges to solve problems with same molecular structure but using different polypeptide sequences. In such cases, sequence based methods often fails and structure based methods are computationally expensive. We show two examples: in one Layers helps to classify same functional protein, lysozyme from two different domains of life; and in another example Layers identify the structurally similar molecules having negligible sequence identity using myoglobin and hemoglobin structures.

**Figure 3:**
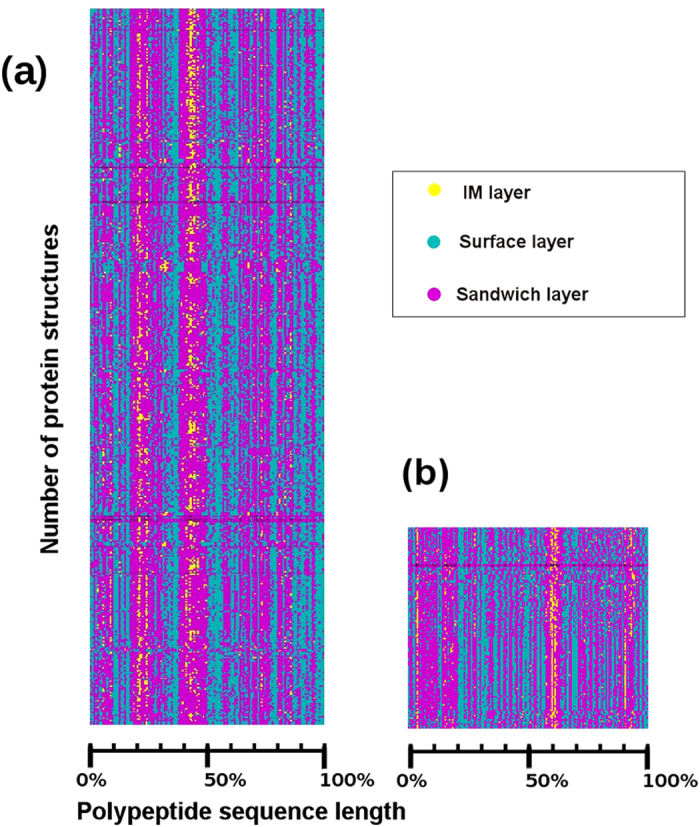
One dimensional RTP of structures stacked together to observe the consensus of residue transition. Relative position of residues in polypeptide sequence is marked along the abscissa, while the number of structures is stacked along the ordinate. (**a**) RTP of eukaryote lysozyme plotted using 542 structures. (**b**) RTP of virus lysozyme plotted using 149 structures.

Lysozyme is the first crystallized enzyme with dynamic properties that has both historical and current interests^26^,^27^. Structures of lysozyme are obtained from the PDB^1^, which are pre-classified as eukaryotes and viruses. If the pairwise sequence identity is very high in a given biological domain, the RTP generated by the Layers is consistent. In contrary, even when there is wide range of sequence identity (Supplementary Fig. 2a) but have similar structures, RTP can be used to identify such proteins (Fig. 3).

Figure 3 represents the RTP of 542 eukaryote (Fig. 3a) and 149 virus (Fig. 3b) lysozymes. The number of structures is shown along the ordinate and the relative sequence length is shown along the abscissa. In eukaryotic lysozymes (Fig. 3a), the first half of the polypeptide sequence is involved in traversing all the layers forming the scaffold, upon which the next half of the polypeptide sequence gets wrapped. In virus lysozymes (Fig. 3b), the IM layer is formed by the residues appearing at the 4%, 60% and 90% of the polypeptide sequence length. Here, the IM layer is formed mainly by the C-terminal residues. Residues in surface and sandwich layers weave in and out of the structures more frequently in viruses than in eukaryotes. This suggests that the long continuous segments are pushed to the surface by eukaryotes, whereas the short discontinuous segments are pushed to the surface by viruses. The RTP generated by Layers is unique and comprehensive to explain the final snap shot of folding pattern of lysozymes from two different domains of life.

Considering the sequence similarity within and between the two classes of lysozyme, it is not straight forward to classify eukaryotic lysozymes based on the pairwise sequence identity, which is very wide (Supplementary Fig. 2a). Slight variations in the RTP within a class are because of the effect of mutations on the tertiary structures. Pairwise sequence identity of virus lysozymes is very high (Supplementary Fig. 2b) and between virus and eukaryotes it is very low (<20%) (Supplementary Fig. 2c). The root mean square deviation (RMSD) for pairwise structural alignment within and between virus and eukaryote lysozymes are presented in Supplementary Fig. 2d–f. Layers generates a consistent RTP for all the structures of eukaryotic lysozymes (Fig. 3a) as their structural similarity is high (≤2.5 Å C-α RMSD) (Supplementary Fig. 2d). The sharp bands in RTP of virus lysozyme (Fig. 3b) can be attributed to high similarity in structures (≤2 Å C-α RMSD) (Supplementary Fig. 2e). The RTP of eukaryotic and the virus lysozymes are identified to be different from each other and unique for each class. This is a computationally inexpensive procedure allowing their classification and identification, which is not possible to identify using simple sequence or structure based methods.

**Figure 4:**
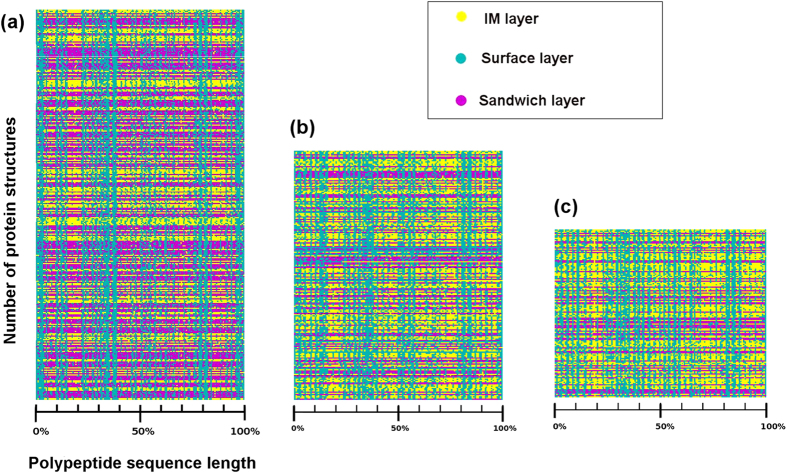
One dimensional RTP of structures stacked together to observe the consensus of residue transition. Relative position of residues in polypeptide sequence is marked along the abscissa, while the number of structures is stacked along the ordinate. (**a**) RTP of myoglobin plotted using 332 structures. (**b**) RTP of alpha chain of hemoglobin plotted using 212 structures. (**c**) RTP of beta chain of hemoglobin plotted using 141 structures.

Layer is used to analyze and gauge its performance with proteins having similar folds but with negligible sequence identity exemplified by myoglobin and hemoglobin structures. Figure 4 shows the RTP of myoglobin, and alpha and beta subunits of hemoglobin. Sequence alignment methods often fail to identify the structurally similar proteins, as exemplified in the case of myoglobin and hemoglobin. We find the RTP generated by layers have similar pattern (Fig. 4) for myoglobin (Fig. 4a) and alpha (Fig. 4b) and beta (Fig. 4c) subunits of hemoglobin even though the pairwise sequence identity between them is less than 30%. Here, the orchestration of residue transition is analogous, resulting in similar folds irrespective of sequence variations.

### Surface sampling for robust and fast shape estimation

Proteins are flexible in nature and fluctuate over an average conformation^28^. Protruding parts form the boundary of a molecule defining its shape. To estimate the shape of the protein molecules, surface atoms may be used while ignoring the atoms present in inner layers. The surface can be further sampled non-randomly preserving the protruding atoms of the molecule. This cuts down the computational load in processes trying to identify molecular shape and surface. Non-random sampling ensures reproducibility, preserves shape features and maintains uniformity among the structures. Non-random sampling through Layers is robust, independent of molecular orientation, structure and shape. Sampling is much different from peeling of layers. Layers peels all the layers sequentially, but sampling works only with the molecular surface. Increasing *Sr* beyond the default value results in non-random sampling.

**Figure 5:**
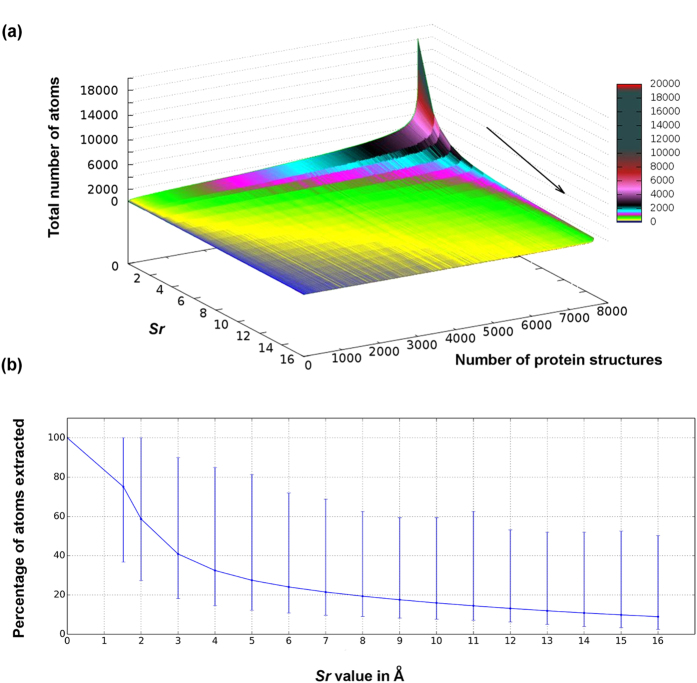
Comprehensive representation of reduction of molecular surface of single polypeptide chain correlated with their total number of atoms and varying *S*_*r*_. This results in sampling of molecular surfaces. (**a**) Magnitude of reduction of atoms due to surface sampling. (**b**) Percentage reduction (with average, minimum and maximum) are given for each *S*_*r*_ (default is 1.52 Å).

**Figure 6:**
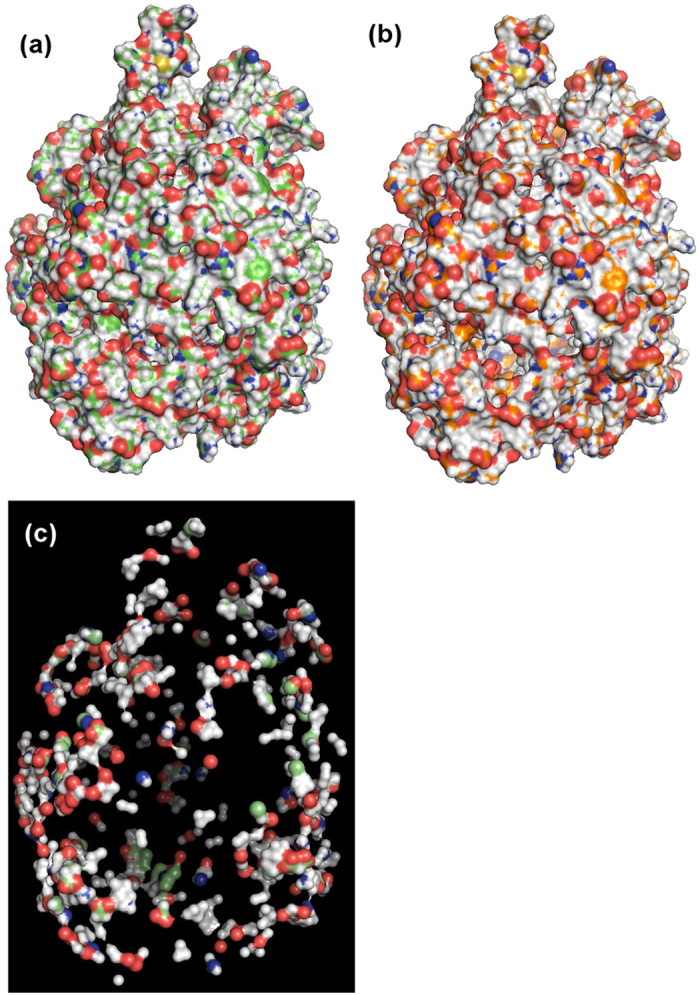
Adopting Layers to generate coarse-grained surface model for a large macromolecule, a metalloprotease with 18,119 atoms (PDB id: 3S5M). (**a**) Actual view of the native structure (with 18,119 atoms). (**b**) Peeled surface layer contains 6655 atoms with a hollow molecular interior. This is a 63% reduction of atoms to represent the same molecular surface while getting rid of internal atoms of the molecule. (**c**) Non-random sampling of surface layer generating a coarse-grain model to further reduce the number of atoms used to represent the surface and the shape of the molecule. This non-randomly sampled surface (with *S*_*r*_ = 16 Å) layer contains only 901 atoms, a reduction 95% of atoms; however, it preserves all protruding atoms to represent the shape features of the molecule.

Figure 5 shows the reduction profile of non-random sampling for 7624 non-redundant single chain polypeptide structures with varying *Sr*. It is obvious that large molecules are reduced with higher magnitude resulting in a steep fall in the number of atoms retained and the reduction is insignificant for small molecules (<200 atoms) (Fig. 5a). Using the default value of *Sr*, an average of 25% of total protein atoms can be reduced (Fig. 5b). Surface sampling reduces the number of atoms exponentially with the increase of *Sr* up to 7 Å, on average losing 80% of total atoms in the dataset. The reduction rate starts reaching a stable state beyond *Sr* = 7 Å, but never reaches to zero. With *Sr* = 16 Å, an average of 90% of total atoms can be removed giving a coarse representation of the molecular surface. In the entire dataset, we achieved a maximum of 63% reduction with *Sr* = 1.52 Å (Fig. 5b). Figure 6a shows the structure of the metalloprotease with 18,119 atoms is the largest structure in the dataset. With the default *Sr*, the extracted surface layer contains 6655 atoms, which is a reduction of 63% of the total atoms present in the molecule (Fig. 6b). However, this reduction does not affect the overall shape of the molecule. With *Sr* = 16 Å, the extracted surface layer is further reduced to 901 atoms, which is only 5% of the total atoms present in the molecule (Fig. 6c).

Progressive reduction of a structure by non-random sampling of the surface layer by incrementally varying *Sr* is shown in Supplementary Fig. 3. Protruding parts of the protein surface that define its shape are extracted for all the *Sr* values, suggesting that the shape of the molecule is not compromised with increased *Sr* (Supplementary Fig. 3b–q). This non-random sampling preserves the global shape of the molecular surface by getting rid of the local protrusions. This provides an acceptable coarse model for a molecule to analyze and identify similar target shape, and is useful to rapidly create a preliminary database of structures with similar shape, that can be further analyzed with stringent features.

## Analysis of Layers

**Figure 7:**
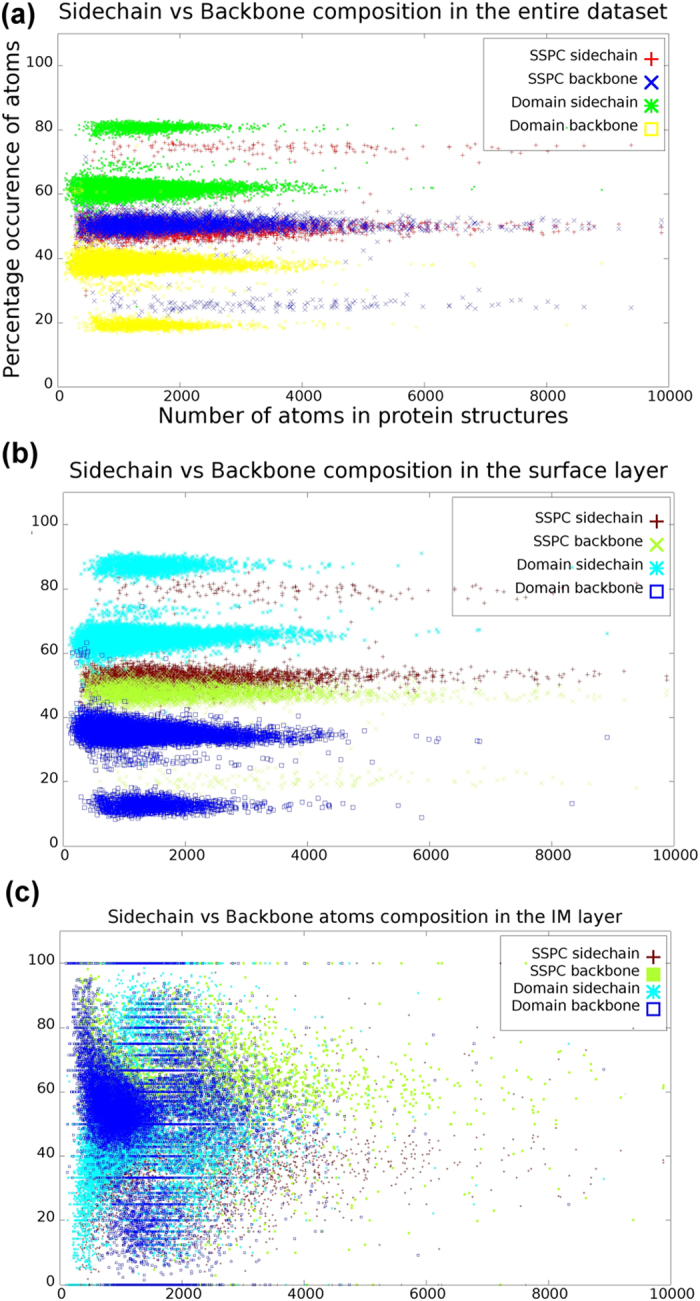
Layers analysis on 7624 structures with single polypeptide chain (SSPC) and 16,983 domains reveal different composition of side chain and backbone atoms in different layers. (**a**) The preference for side chain and backbone atoms is distinct in domain structures, where they are almost similar in SSPC. (**b**) Preference for side chain atoms increases in both SSPC and domains in surface layer. (**c**) IM layer showing preference for backbone atoms in SSPC and domains.

Two different datasets are used to show layers analysis of protein structures. One is the dataset based on the domain classification of CATH^29^, and the other is the dataset of structures with single polypeptide chain (SSPC) collected from PISCES^30^. As missing residues in the structures may introduce noise in the RTP, structures with less than 5% missing residues are used in this analysis. If the IM layer contains atoms from single amino acid residue then it merged with its upper layer and is used as the IM layer as single residue is insignificant for interactions. The following analysis helps to validate the Layers algorithm proving some known facts about proteins and also emphasizing some new features of proteins explained in terms of layers. Layers obtained similar results for SSPC and domain datasets (Fig. 7 and Supplementary Fig. 4 and 5).

Supplementary Fig. 4 shows the residue composition in the IM layer and in the surface layer. The IM layer of SSPC is dominated by nine residues, whereas the surface layer is dominated by ten residues leaving Thr almost equally found in both the layers and in both SSPC and domain datasets. As expected, while the hydrophilic residues are abundant at the surface layer, hydrophobic and aromatic residues are dominant at the IM layer. Besides, Gly and Pro are found more frequently at the surface layer than at the IM layer, Gly showing strikingly high percentage in domains and Pro in SSPC dataset. With its smallest side chain, Gly can facilitate the conformational flexibility of the surface. Owing to the restriction of the side chain flexibility, Pro is not preferred at the well packed protein core.

The distribution of the atoms in the surface and in the IM layers is shown in Supplementary Fig. 5. The number of atoms in the surface layer increases linearly with the size of the protein in both SSPC and domain datasets, whereas the number of atoms in the IM layer is constant irrespective of the size of the protein (Supplementary Fig. 5a). The constant number of atoms in the IM layer may be justified by the packing density of the protein core, which is comparable with the crystals^31^. Numbers of residue constitute the IM layer shows bifurcated clusters: one is sharp and linear, while the other is scattered (Supplementary Fig. 5b). A maximum of 25% of total atoms in a structure are found in the IM layer and a minimum of 40% of total atoms are found in the surface layer (Supplementary Fig. 5c).

One may presume that the number of layers should increase with the size of the protein; however, it is delusive (Supplementary Fig. 6). Majority of the proteins are found to have three layers irrespective of their size. Proteins having residues between 30 and 160 are generally peeled into two to four layers (Supplementary Table S2), while the proteins having residues more than 160 but less than 450 peeled into two to five layers. Proteins having residues more than 450 are generally peeled into three to four layers, and very often into five layers.

Proteins are expected to communicate through their surface, thus exposure of the side chain atoms on the surface is essential. Figure 7 compares the backbone (N, CA, C and O) and the side chain (all the atoms except the backbone atoms) composition of the amino acid residues in the entire dataset viz-a-viz in the surface and in the IM layer. In the SSPC dataset, the side chain and the backbone contribute almost equally except in some structures where the side chain dominates (Fig. 7a). In contrary, the domain dataset shows a striking preference for the side chain atoms. However, the analysis through Layers reveals that the surface layer is dominated by the side chain atoms (Fig. 7b), while the IM layer is dominated by the backbone atoms (Fig. 7c) in both SSPC and domain datasets. This observation can be interpreted as the backbone atoms are pushed into the core of the protein exposing the side chain atoms to the periphery, facilitating the wrapping of the next layer that may define the affinity and selectivity for the residues in the next layer.

### Prediction of RTP for a given protein sequence

**Figure 8:**
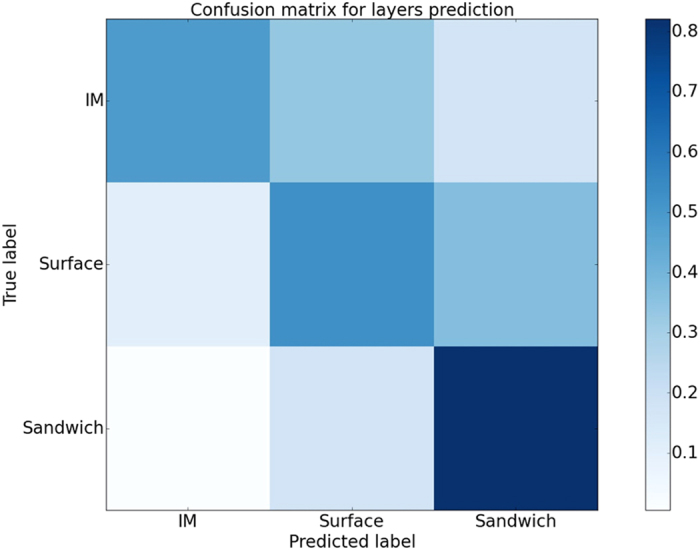
Confusion matrix shows the classification accuracy of the prediction model based on random forest. Diagonal values represent the correct predictions, whereas off diagonal values are incorrect predictions. Each cell represents the normalized accuracy for each class.

We have extended our analysis to design a classifier to predict the RTP for a given amino acid sequence. We have trained our model using random forest on a dataset of 16,983 protein domains derived from CATH^29^. Figure 8 shows the normalized confusion matrix, which represent an average of 65% overall prediction accuracy. Diagonal values in the confusion matrix represent the correct predictions, whereas, off diagonals represent the wrong predictions. We have used three classes: IM, Surface and Sandwich layers. The sandwich layers are predicted with more accuracy than the other two classes. In all the three classes, majority of false predictions are close to their actual classes. Though the model is not highly accurate, it is our first step towards generating a biased initial configuration for protein folding simulations.

### Comparison of surfaces extracted using Layers and SASA method

The SASA of a protein can be calculated using the program Naccess^21^, which follows Lee and Richards algorithm^32^. In general, a residue is considered on the protein surface if its relative accessibility loss is ≤5%^33^. Here, we present an analysis to emphasize the differences in molecular surface extraction using Layers and SASA method (Supplementary Fig. 7). Residues on the surface need to be extracted as a whole using SASA; whereas, Layers extracts atoms that are part of a residue. Because of the different approaches by SASA and Layers the total number of atoms and residues extracted as surface vary. Supplementary Fig. 7 shows that both perform close to each other with few exceptions. The number of residues identified in the surface by Layers is always higher than that of by SASA. This can be attributed to the fact that Layers extract every single atom that appears on the surface. The total number of atoms extracted as surface by the Layers is less compared to the SASA.

## Discussion

We have developed Layers, which identifies residue transition pattern from folded protein structures, extracts surface atoms and non-randomly samples the molecular surface. We have also introduced RTP, a methodology inspired from the nucleation mechanism of protein folding^20^. The RTP based on peeled layers can be used to identify proteins with similar folding patterns. Layers can be merged with different algorithms to reduce computational load and can generate coarse models of molecular shape at tunable fineness.

RTP generated by Layers is consistent even if there are large deviations in pairwise sequence identity. The conserved RTP proves that Layers is insensitive to rotation and translation variants of molecules in 3D space (Fig. 3a). Instead of using entire molecule, surface layer peeled using Layers can be used to identify similar molecules, which can be used as drug targets^34^. The RTP shows that the nucleus or the scaffold is dominated by the residues at the N-terminal of the polypeptide chain in eukaryote lysozymes, whereas in viruses, it is dominated by the C-terminal residues. In eukaryotes, the first half of the polypeptide contributes to the formation of the core of the structure (Fig. 3a) upon which the next half wraps that involved in the recognition of antibody^35^. This may imply that the folding of lysozymes in eukaryotes is initiated from the N-terminal and leaves the C-terminal for communicating with its partners. With RTP we identified the different folding patterns for eukaryotic and virus lysozymes.

Surface and shape properties of proteins are critical in determining its biological activity, and are successfully used by many applications in molecular docking, drug designing and local similarity identification^36^,^37^,^38^,^39^. Macromolecular docking can be used to identify protein partners, screen drug targets, model macromolecular interfaces and engineer interfaces with novel complexes exhibiting desired functions^38^. Many applications including molecular docking work with molecular surface^21^,^32^,^40^; and instead of using the entire molecule, surface layer extracted by Layers can be used to reduce the computational load making it feasible to work with large databases.

Layers performs non-random sampling of the molecular surface, while preserving the overall molecular shape. The number of atoms in a structure exponentially decreases with increased *Sr,* which can be tuned to sample the molecular surface. Protruding atoms of a molecule have great biological significance; they are part of the active sites, protein epitopes and interfaces in molecular complexes. Thornton *et al.*^41^, showed that the antigenic determinants of proteins are present at the protruding parts of the protein. Volumetric methods used to identify active sites through surface cavities consider the protruding atoms of protein^42^,^43^,^44^,^45^. Target properties on the surfaces can be optimally aligned by using surface layers only instead of the entire molecule. Local similarity search on the surface layers will be faster and reliable as active sites are most often located on the molecular surface.

IM layer represents the center of the molecule and is equivalent to the core of the protein. The constant percentage of total atoms and bifurcated clusters for the number of residues forming the IM layer are comparable to the packing density of proteins^31^. Molecular weight (M) of a protein shows clear influence on packing density^46^. Proteins with M <20 KDa shows densely packed protein interior, and this density reduces with the increase of M^46^. These limits are in coherence with the sharp linear cluster and scattered clusters obtained by Layers reflecting the dependency of packing density on molecular weight (Supplementary Fig. 5b). Analysis on molecular composition of the side chain and the backbone atoms shows that a subset of our dataset has clear abundance of side chain atoms (Fig. 7a). Side chain and back bone composition in the surface layer shows a noticeable drift from the general trend shown by the protein structures in SSPC dataset. However, domains show an innate preference for side chain atoms (Fig. 7a). On the contrary, the IM layer is dominated by the backbone atoms rather than the side chain atoms in both SSPC and domain datasets. The contrasting behavior of the IM layer can be justified by its nature to facilitate the folding process. The side chain atoms are exposed to the upper layer that can effectively guide the neighboring residues to bind and may help to the progress of the folding.

Layers extracts more number of residues and less number of atoms as surface compared to the method based on SASA (Supplementary Fig. 7). Loss of relative accessibility by a residue does not mean that its every atom is on the surface of the molecule. Similarly, atoms can be on the molecular surface even if the residue to which the atom belongs has relative accessibility below a certain cut-off. This results in a less number of residues and more number of atoms identified on the surface by SASA method, and it is ineffective to extract all surface atoms besides losing the atoms that are not actually on the molecular surface.

Layers generates the folding pattern of a given molecule, which can be used to study folding unfolding simulations. Protein folding simulation using biased initial configuration is successful and can reduce the simulation time up to 99%^47^. We used random forests to classify the residues of primary sequence into IM, surface and sandwich layers for which we achieved an accuracy of 65%. This low accuracy can be explained as observed in the Fig. 4. Here, only the surface layer is showing striking matches in all three structures, whereas the other layers are noisy and not distinct. The lower prediction accuracy is may be because of these kinds of noisy layers. Improved prediction model can be used to generate biased initial configuration. This provides a scope that our algorithm and methodology can help to accelerate protein folding simulation experiments by providing biased initial configuration.

## Online Methods

### Algorithm

Euclidian distance between any two atoms is calculated using the equation 1. Three cylinders are defined for every atom of a protein structure by using equation 2, and each cylinder is oriented along the *x, y* and *z* axes of 3D space. As of cylinder oriented in *x-axis*, the *x* coordinates are considered zero for calculating their distance with other atoms (eq. 3). The reference atom and its distance with all other atoms is calculated using *y* and *z* coordinates. All those atoms with distance less than *Sr* are collected into the cylinder. Atoms with maximum and minimum value for *x* coordinate are selected as a layer (eq. 4). For *y* cylinder (eq. 5, 6), all the atom’s *y*-coordinates are considered as zero, and distance from reference atom are calculate using *x* and *z* coordinates. Atoms with distance less than *Sr* are collected into *y* cylinder and among these collected atoms the atoms with maximum and minimum value for *y* coordinate are selected as a layer. For *z* cylinder (eq. 7,8), all the atom’s *z*-coordinates are considered as zero, and distance from reference atom are calculate using *x* and *y* coordinates. Atoms with distance less than *Sr* are collected into *z* cylinder and among these collected atoms, the atoms with maximum and minimum value for *z* coordinate are selected as a layer. Now the reference atom changes from *i* to *i* + 1. This process iterates for all N atoms present in the structure. After iterating for N atoms, a single layer gets labeled and the layer is numbered according to the iteration on the molecule (eq. 9). Iterations on atoms must not be confused with iterations on molecule. Atoms that are not selected into the current layer (eq. 10) are subject to next iteration for extracting next layer. Iteration continues as long as no atoms are left unassigned. The final layer extracted is considered as the IM layer.


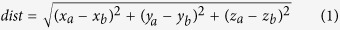



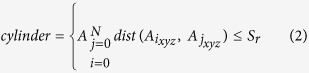



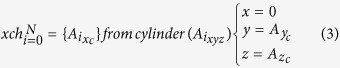



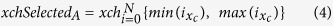



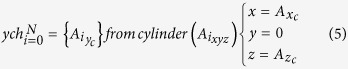



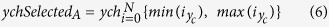



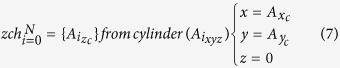



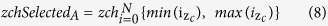



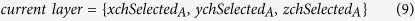



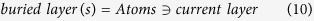


Where *x*_*c*_*, y*_*c*_*, z*_*c*_ are *x, y* and *z* coordinates, respectively; *A*_*xc*_, *A*_*yc*_, *A*_*zc*_ are *x, y, z* coordinates of atom ‘A’, respectively; *xch*, *ych*, *zch* are cylinders along *x, y, z* axis, respectively; *S*_*r*_ is the smallest atomic radius.

### Dataset

We have used two different datasets to extract layers using Layers. One is the protein domain dataset downloaded from CATH^29^, which contains 16,983 domains with sequence identity ≤40%. Other is the structures with single polypeptide chain (SSPC) dataset obtained from PISCES^30^. PISCES^30^ is used for selecting a non-redundant dataset of 7624 crystal structures of single polypeptide chain with pairwise sequence identity ≤25% and resolution better than 3.0 Å. Three dimensional X-ray structures of the selected polypeptide chains were downloaded from the PDB^1^.

### Sequence and structure comparison

Clustal omega^48^ is used to perform pairwise sequence alignment (Supplementary Fig. 2a–c). Pairwise structural alignment (Supplementary Fig. 2d–f) is performed using g_rms package available with Gromacs v4.6.5^49^, which is used to calculate the root mean square deviation for the provided structures using least square fit method for C-α residues.

### Random Forest based prediction model

Random Forest^50^ classification is implemented using WiseRF provided with Anaconda package for python and scikit-learn library of python. Layers are divided into three classes: IM, Surface and Sandwich. We have used 49 different physico-chemical properties of amino acid sequences, with a 13 residue sliding window method. Prediction model is generated using 200 trees, beyond which there is no increase in prediction accuracy.

### Residue transition pattern (RTP)

Polypeptide sequence is superimposed with layer information obtained from the tertiary structure. RTP is represented in both 2D and in 1D. In 2D pattern, comprehensive information of a molecule is provided; whereas, 1D pattern can be used to perform large scale comparisons. The multiline 2D pattern is transformed into a multicolor 1D pattern, with color coding designated to layers. Unlike 2D pattern, 1D pattern represents the relative position of a residue in a polypeptide sequence.

## Additional Information

**How to cite this article**: Karampudi, N.B.R. and Bahadur, R. P. Layers: A molecular surface peeling algorithm and its applications to analyze protein structures. *Sci. Rep.* **5**, 16141; doi: 10.1038/srep16141 (2015).

## Supplementary Material

Supplementary Information
