# Supplementary material for: Layers: A molecular surface peeling algorithm and its applications to analyze protein structures
Source: Sci Rep. 2015 Nov 10;5:16141. doi: 10.1038/srep16141 (PMC4639851; doi:10.1038/srep16141)
Supplement: Supplementary Information [file srep16141-s1.doc]

Layers: A molecular surface peeling algorithm and its applications to analyze protein structures

Naga Bhushana Rao .K^1^, Ranjit Prasad Bahadur^2*^

1. Advanced Technology Development Center, Indian Institute of Technology Kharagpur, Kharagpur-721302, West Bengal, India.
2. Computational Structural Biology Lab, Department of Biotechnology, Indian Institute of Technology Kharagpur, Kharagpur-721302, West Bengal, India.

*To whom correspondence may be addressed

E-mail: r.bahadur@hijli.iitkgp.ernet.in

or

[ranjitp_bahadur@yahoo.com](mailto:ranjitp_bahadur@yahoo.com)

Phone: +91-3222-283790

Fax: +91-3222-27870

**Supplementary Figure Legends**

Supplementary Figure 1 | Primary structure of the Barnase (PDB id: 1A2P) associated with the RTP obtained from peeled layers. Colour scheme is according to Figure 2. Total number (N) and percentage composition of residue types are shown in the bottom right panel.

Supplementary Figure 2 | Distribution of pairwise sequence identity and structural similarity of lysozymes. Distribution of sequence identity (a) within eukaryote, (b) within viruses and (c) between eukaryote and virus lysozymes. Distribution of structural similarity (d) within eukaryote, (e) within viruses and (f) between eukaryote and viruses.

Supplementary Figure 3 | Coarse grain molecular surface model of CRD of human Galectin 3 (PDB id: 3ZSJ) generated by non-random sampling of surface by varying *Sr*. (a) Native structure of the molecule. (b-q) Coarse grain model sampled at different *Sr*.

Supplementary Figure 4 | Amino acid composition in the IM and in the surface layers of SSPC and domains.

Supplementary Figure 5| (a) Distribution of atoms and residues in the IM and in the surface layers. (b) Distribution of residues in the IM layer. (c) Percentage of atoms found in the IM and in the surface layers.

Supplementary Figure 6| Distribution of layers in SSPC and in domain datasets.

Supplementary Figure 7 | Comparison of Layers and SASA methods for identification and extraction of surface residues and surface atoms for some randomly selected protein structures.

**Supplementary Table Legends**

Supplementary Table 1 | Distribution of residue composition in Barnase (PDB id: 1A2P). Supplementary Table 2 | Number of layers and their respective lower and upper bounds of number of residues and atoms in the SSPC dataset.

**Supplementary Figure 1**

**
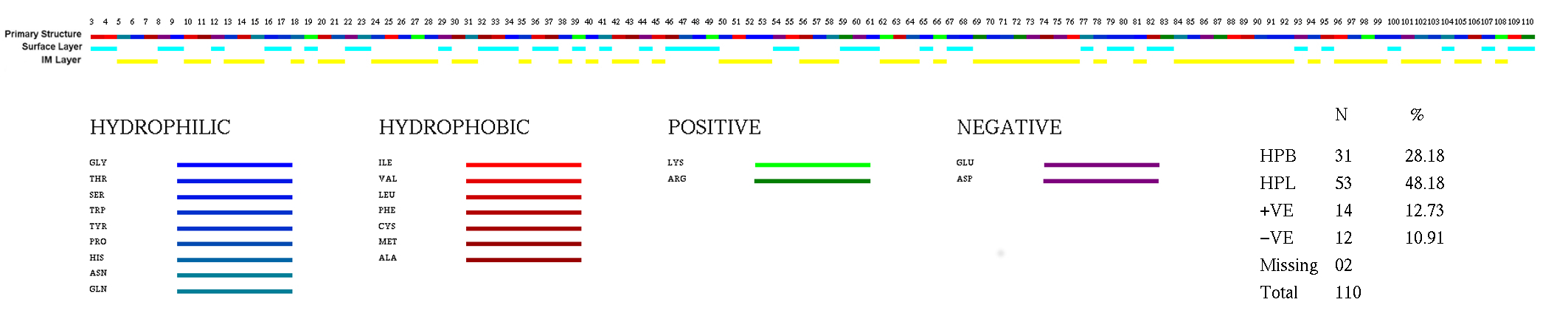
**

**Supplementary Figure 2**


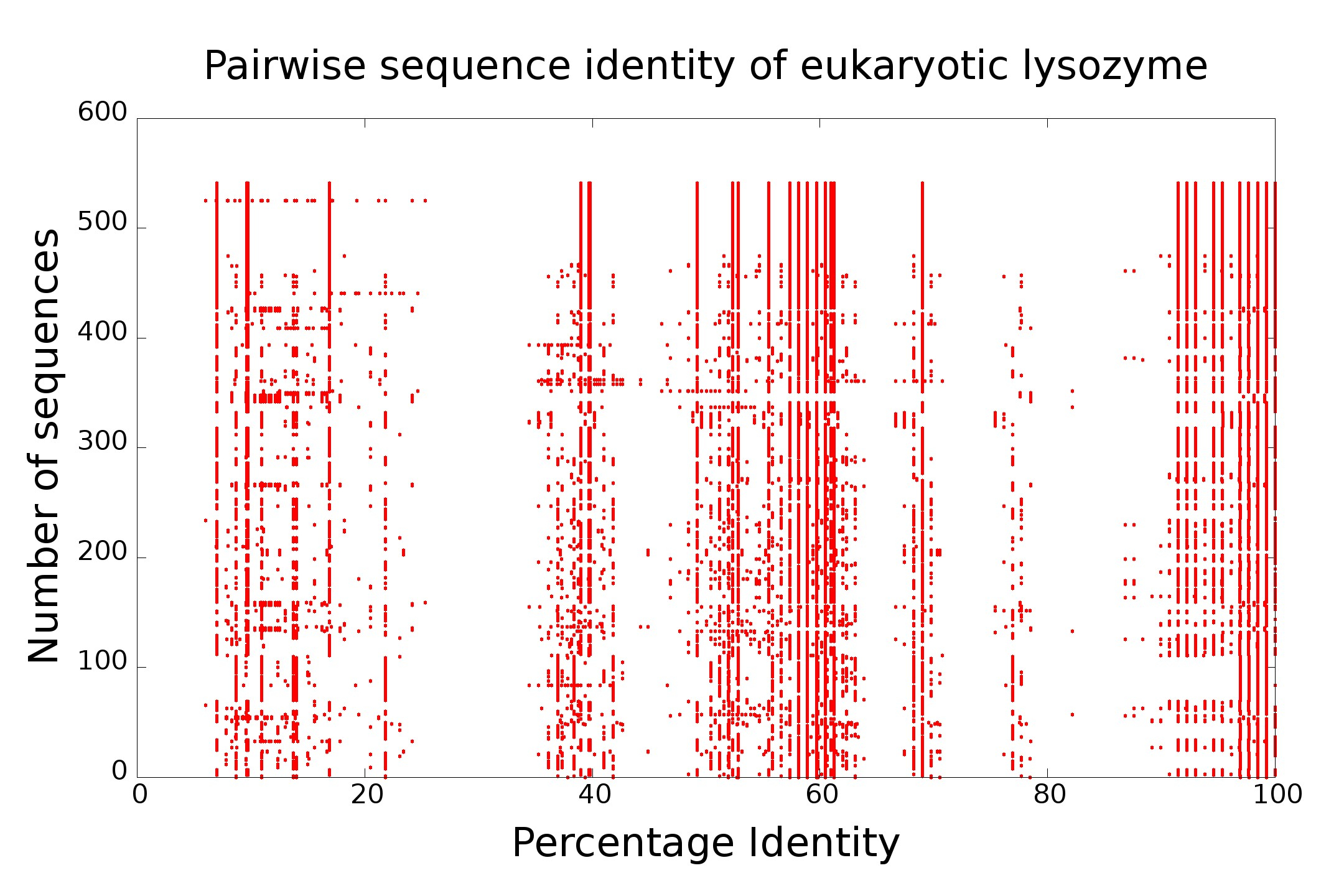
**a**

**
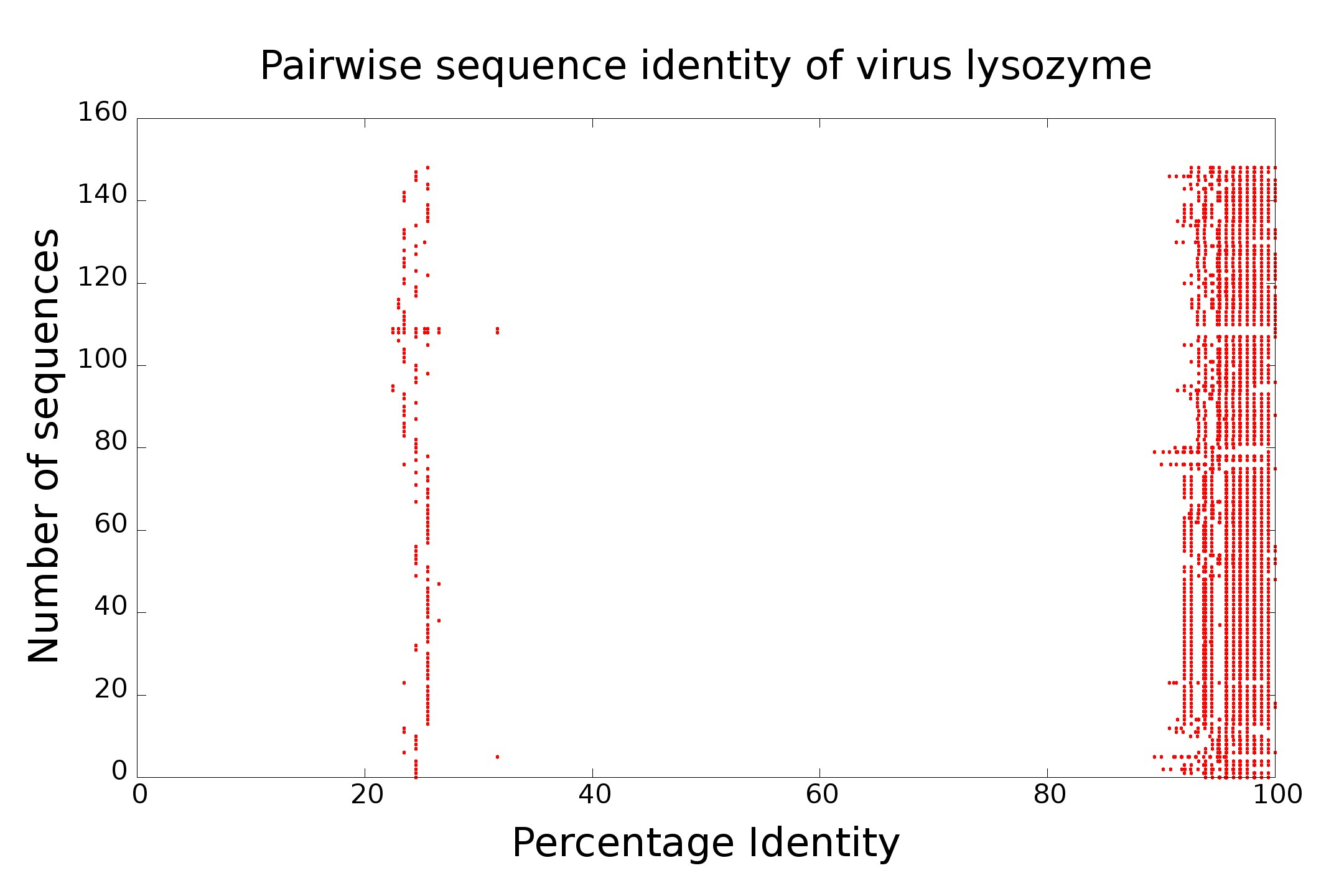
**

**b**


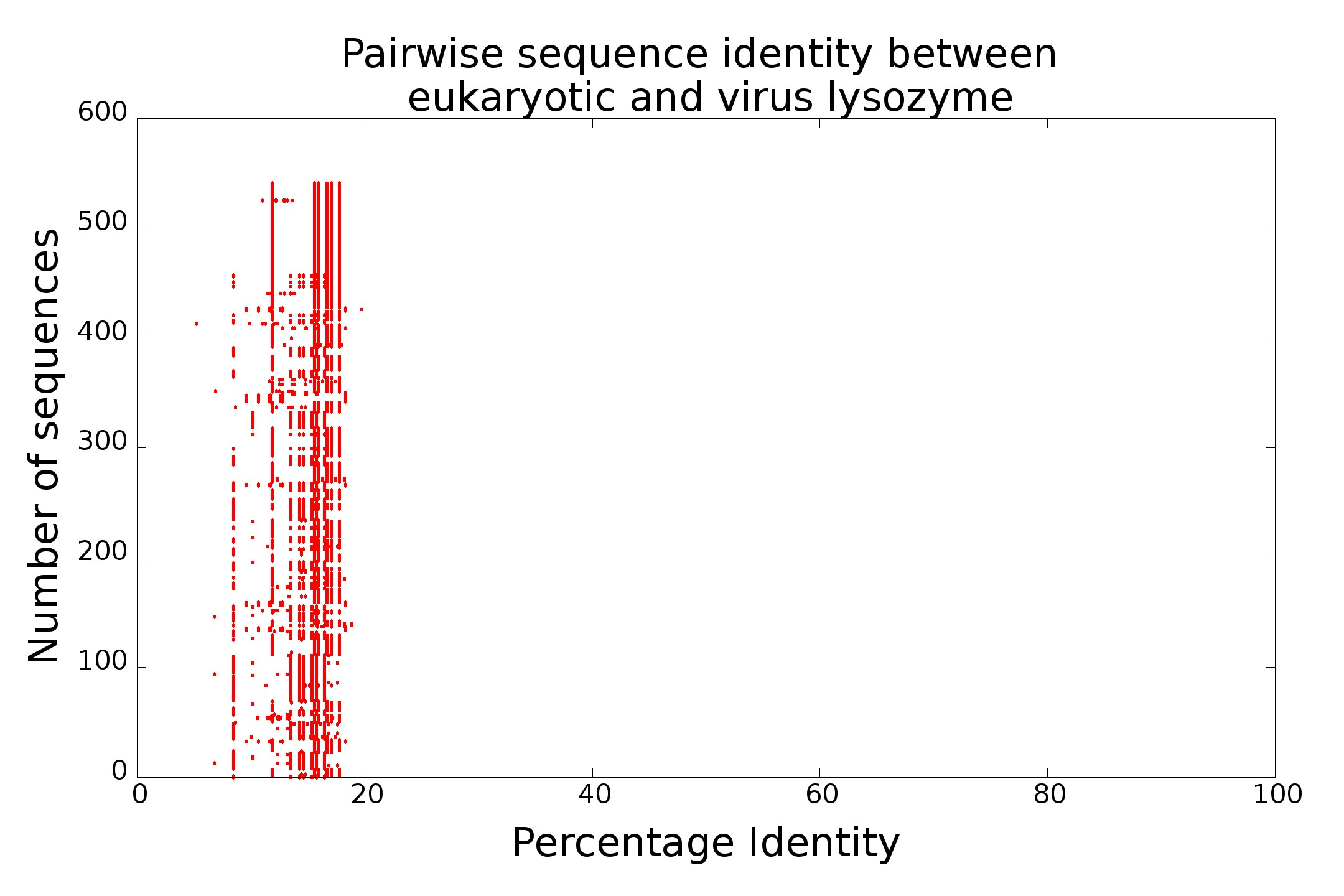
**c**

**d**

**
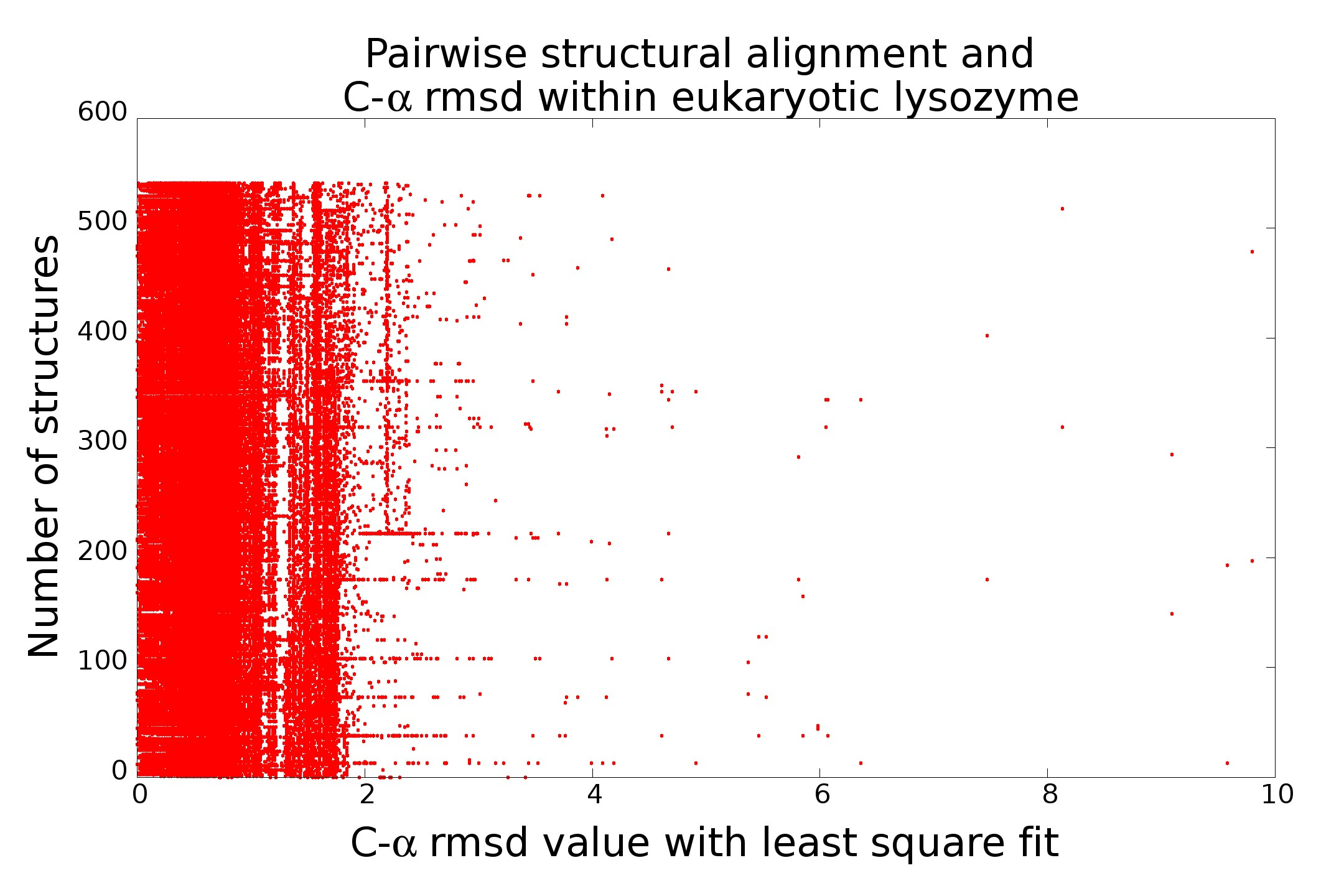
**

**e**

**
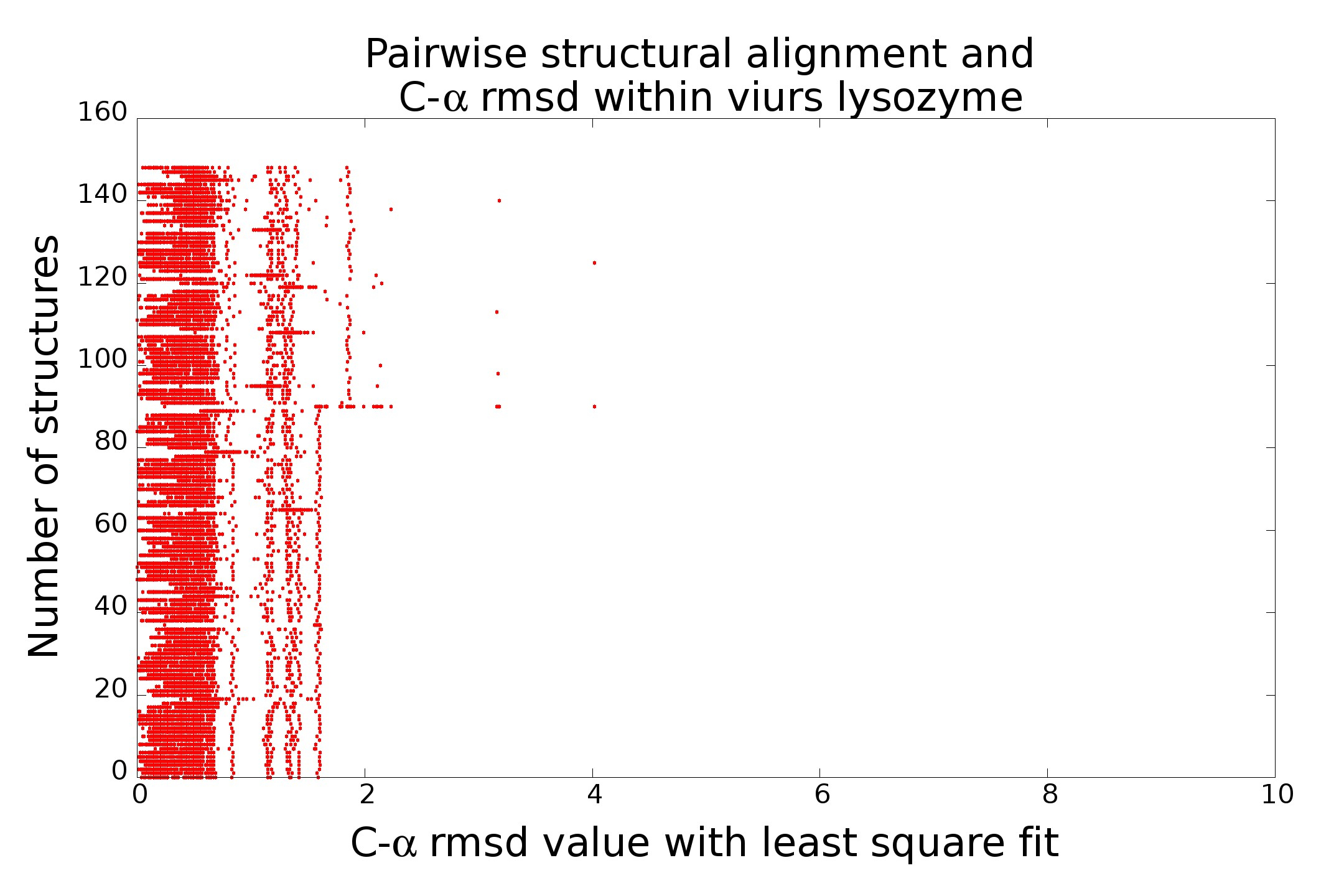
**

**f**

**
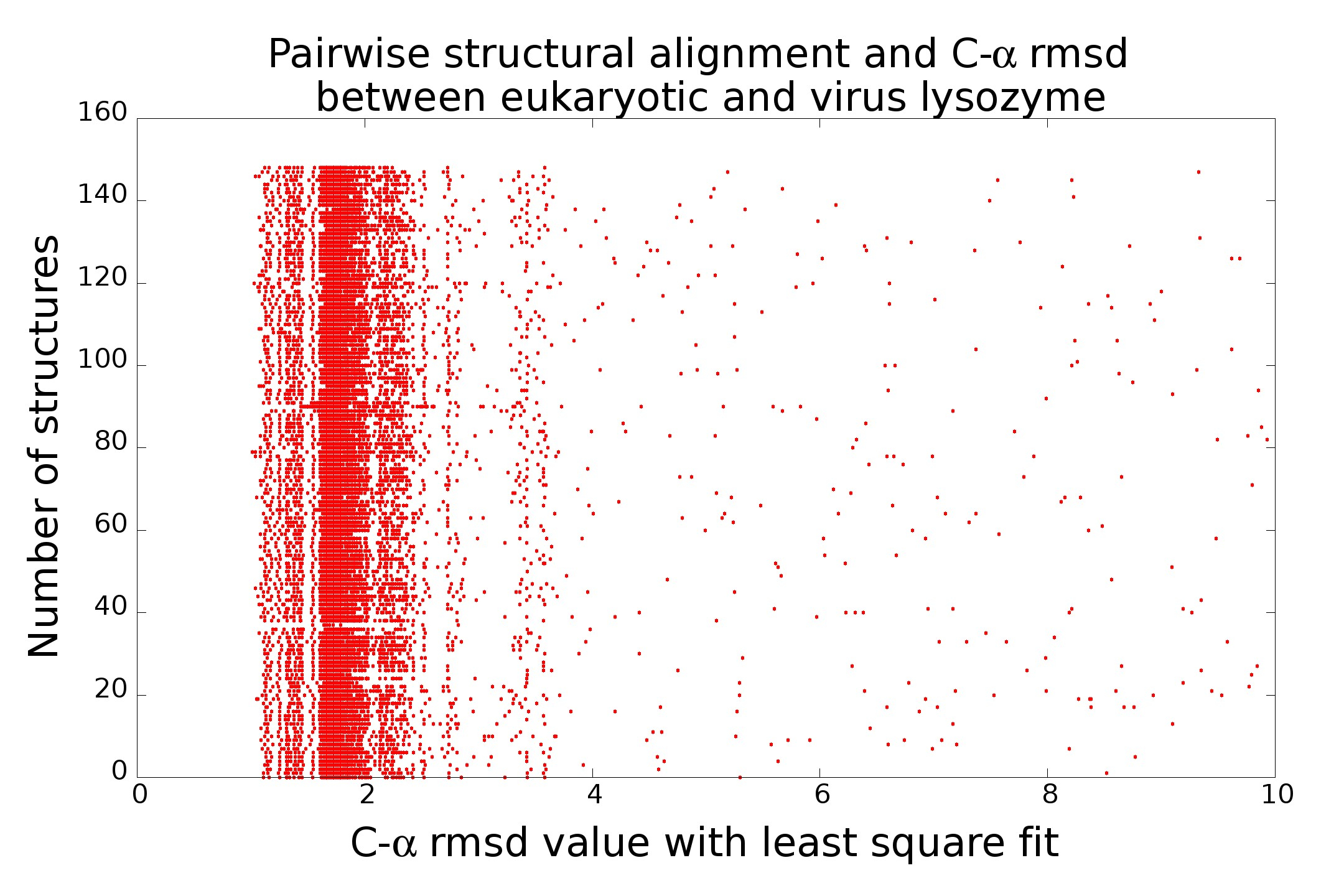
**

**Supplementary Figure 3**


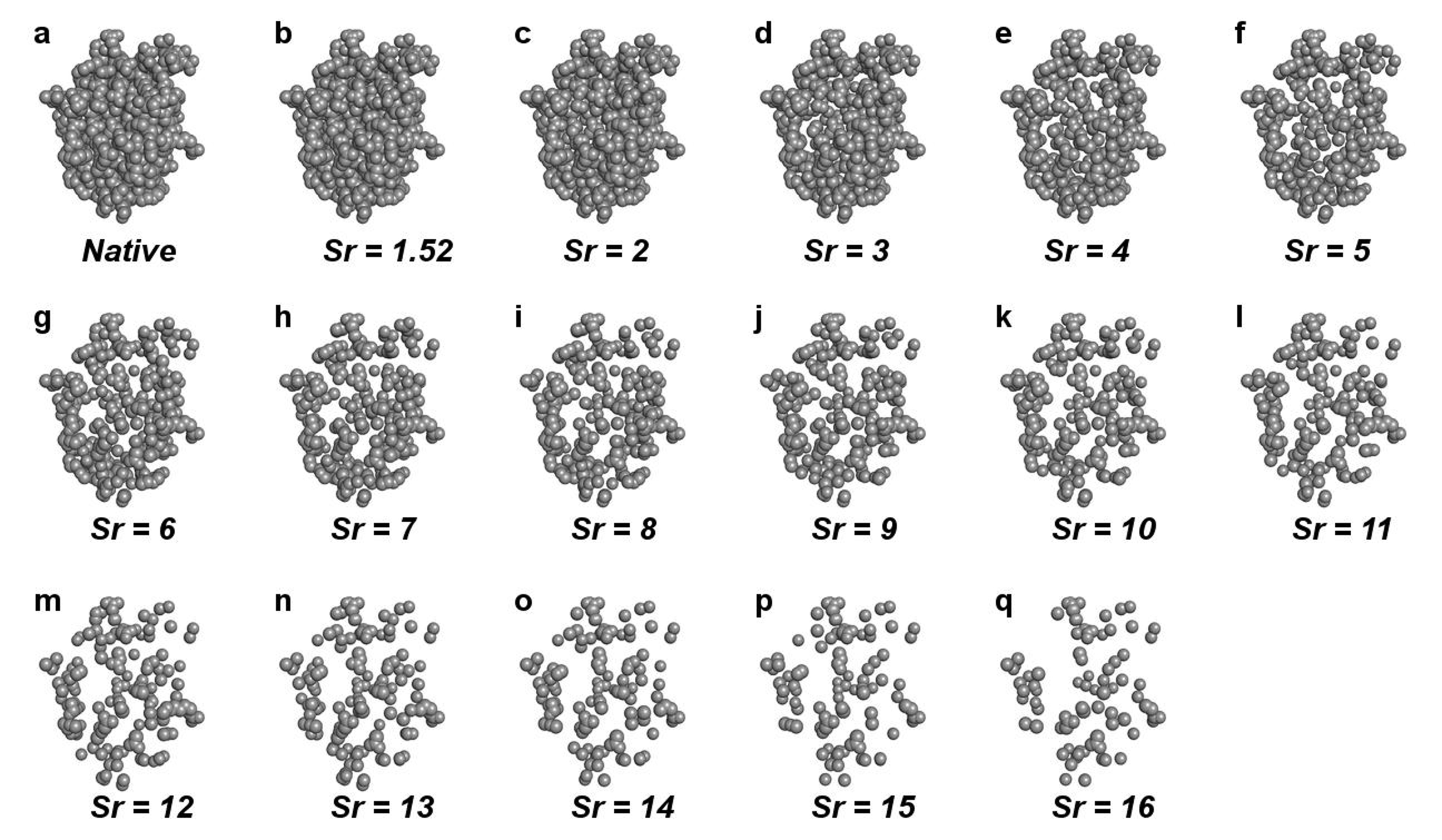


**Supplementary Figure 4**


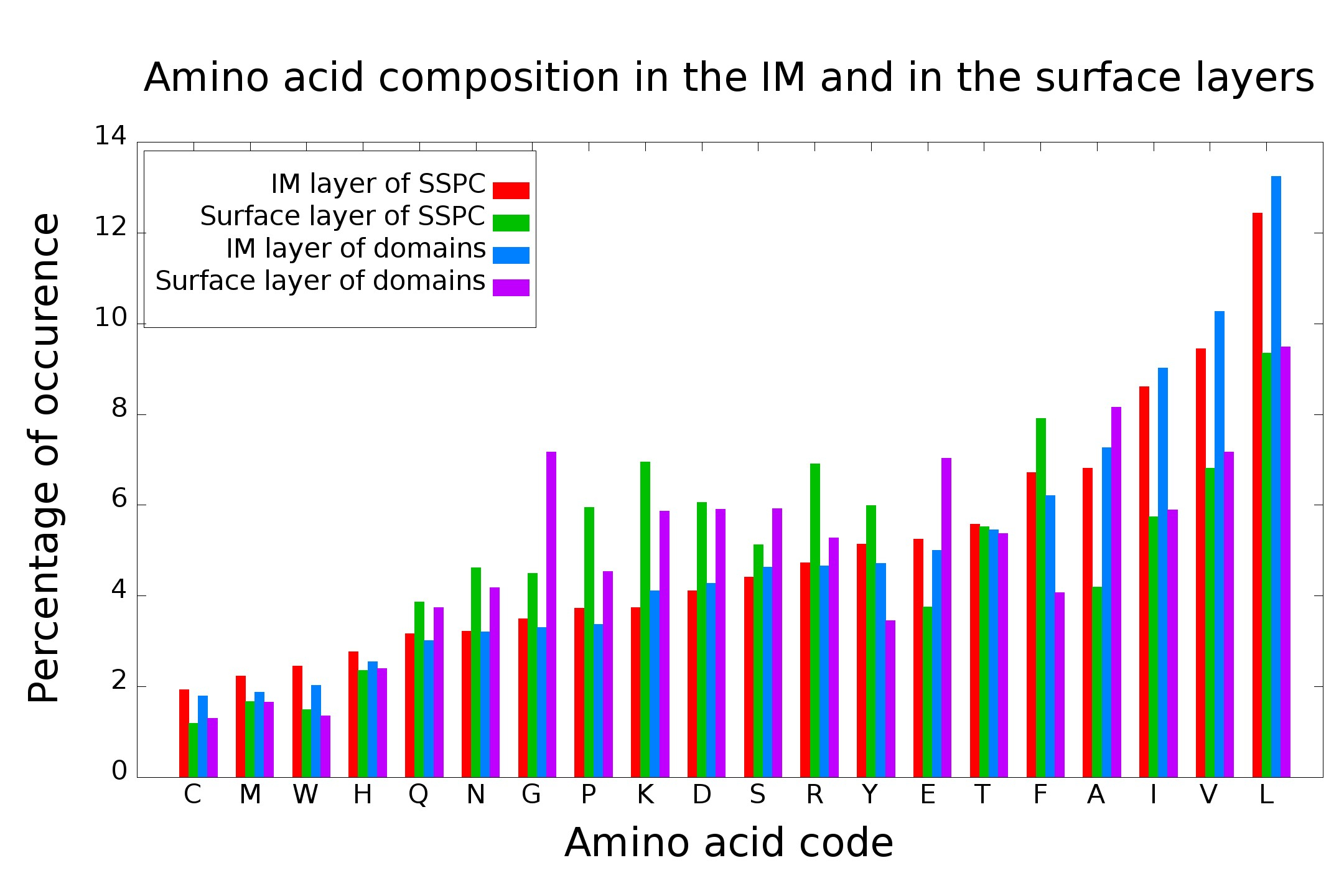


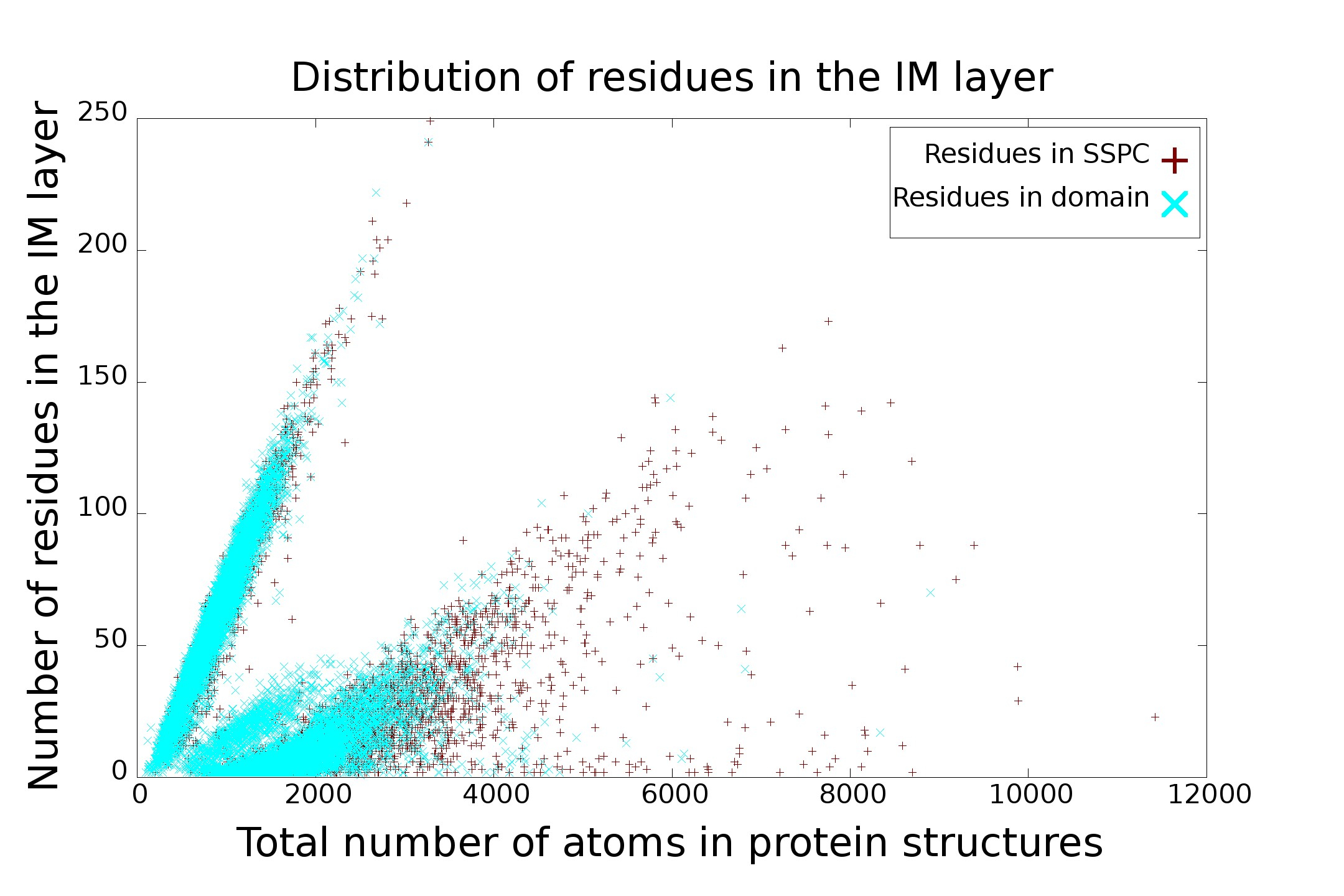
**Supplementary Figure 5**


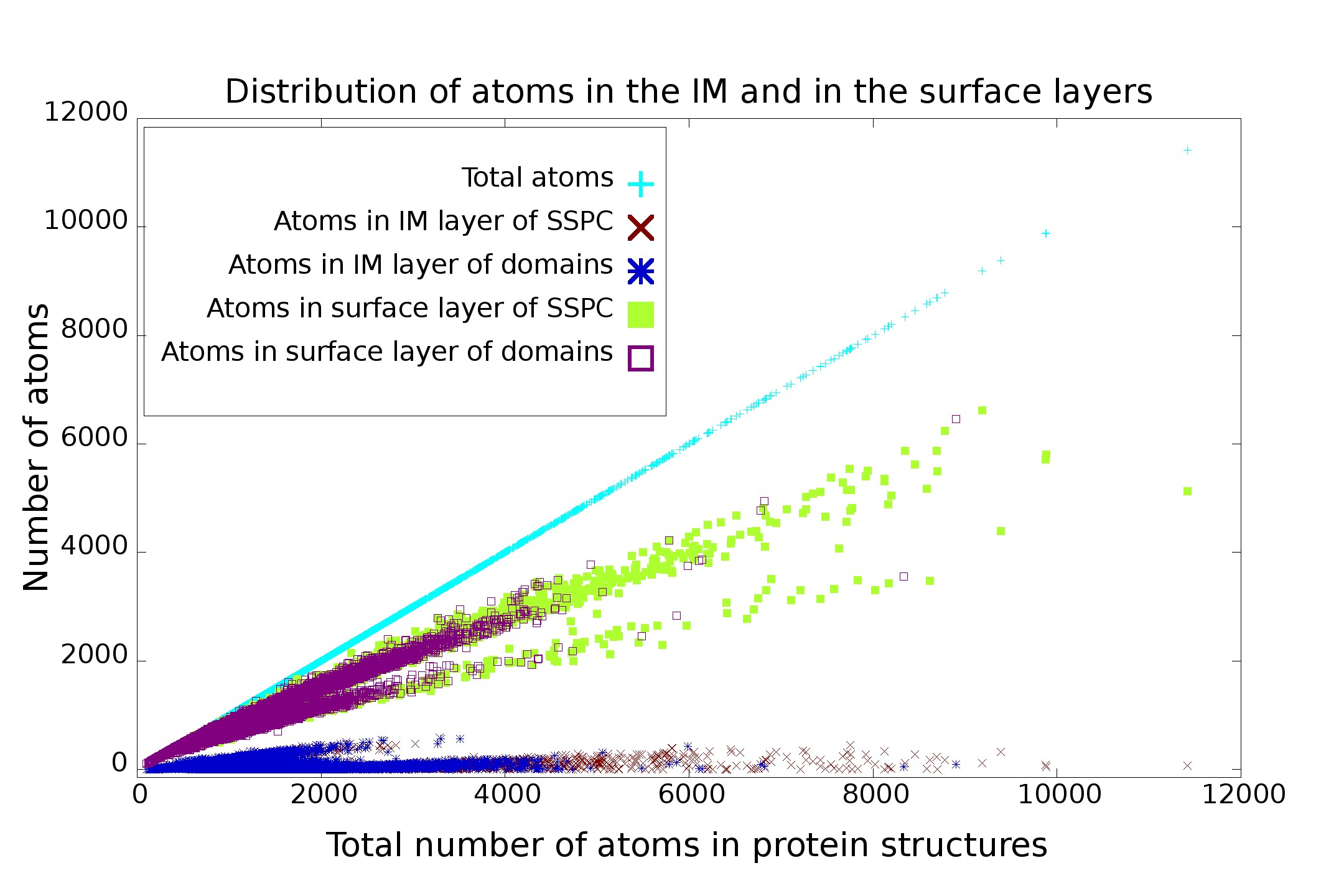
**a**

**b**

**c**


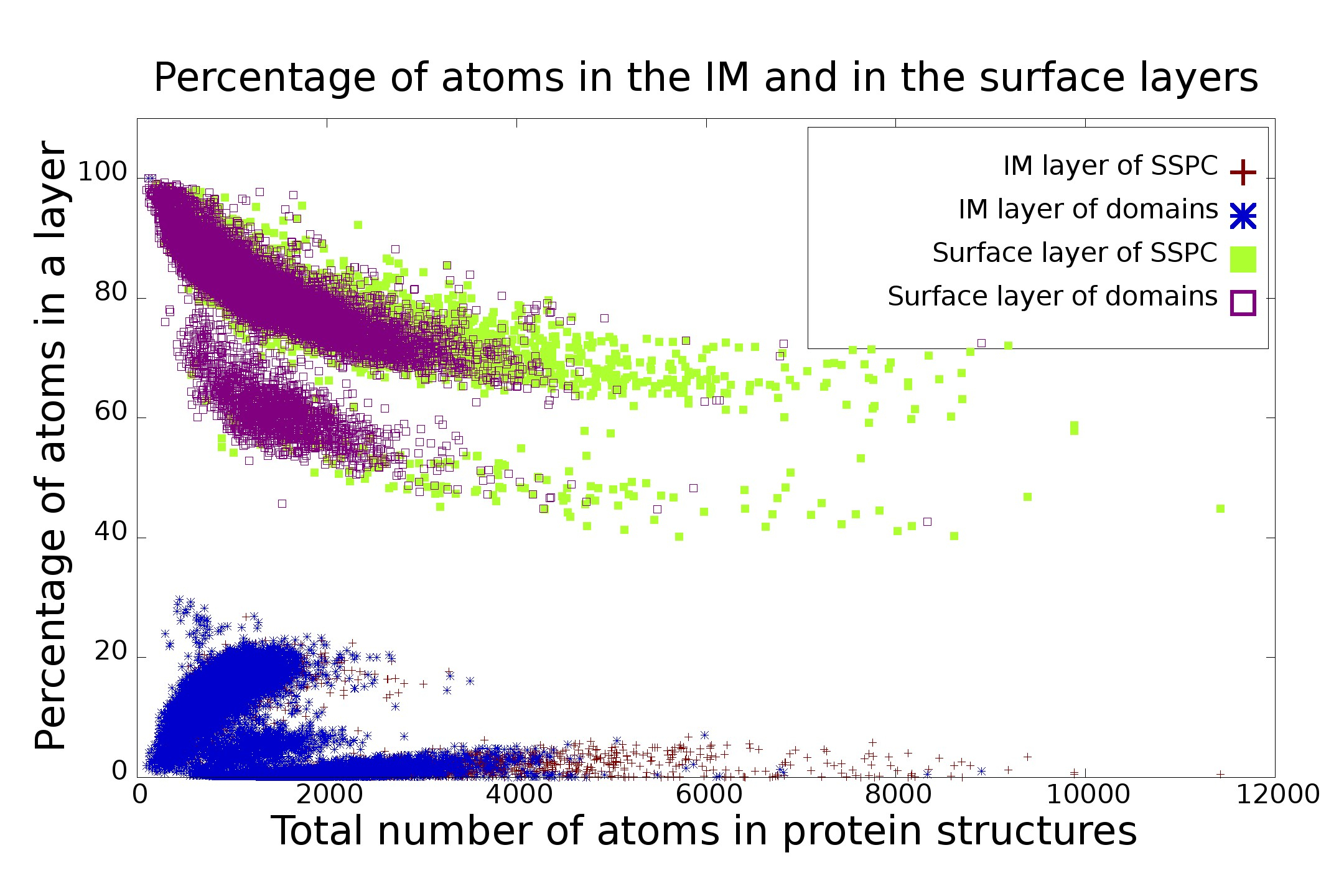


**Supplementary Figure 6**


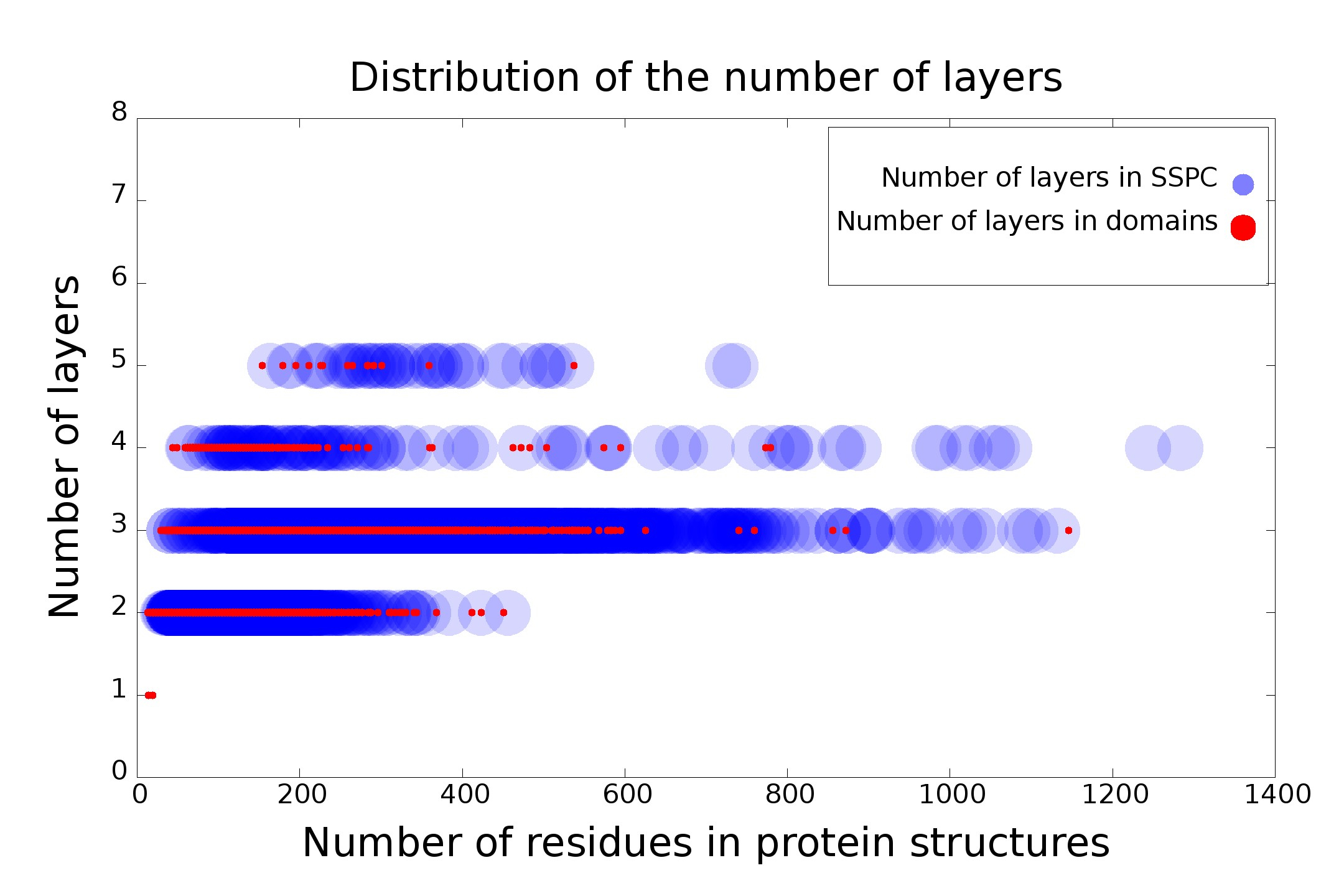


**Supplementary Figure 7**


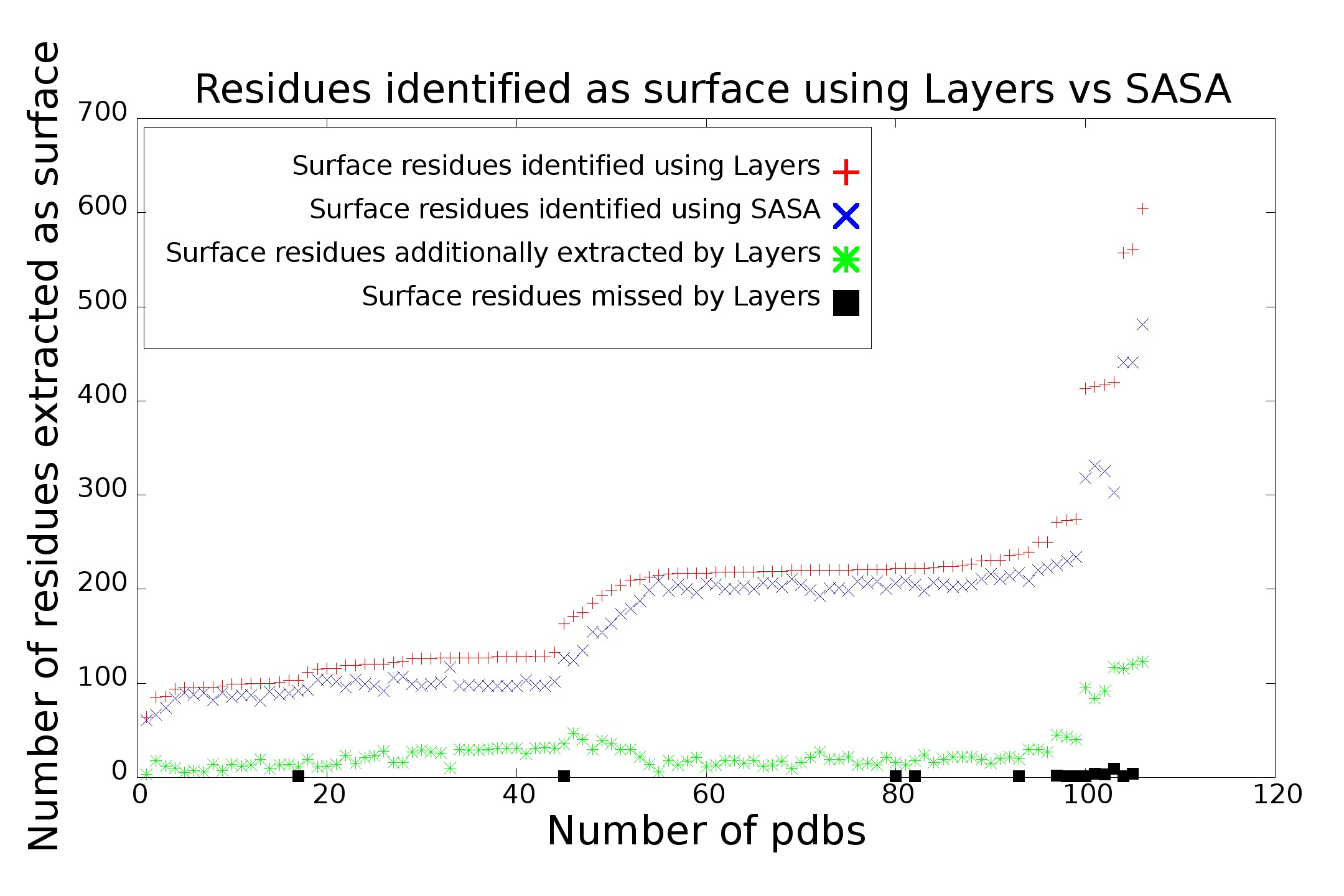


**Supplementary Table 1**

| Residue type | % of residues | | Propensity^a^ of residues | |
| --- | --- | --- | --- | --- |
|  | surface layer | IM layer | surface layer | IM layer |
| Negative | 18.6 | 6.2 | 0.51 | -0.60 |
| Positive | 14.0 | 12.3 | 0.08 | -0.05 |
| Hydrophilic | 41.9 | 52.3 | -0.14 | 0.08 |
| Hydrophobic | 25.5 | 29.2 | -0.08 | 0.50 |

Classification is based on the residue type and their absolute and relative occupancy in surface and in IM layers.

^a^Propensity = ln[(Number of residues of type X in a particular layer/Total number of residues in that layer) / (Total residues of type X in the structure/Total number of residues in the structure)]

**Supplementary Table 2**

| Layer number | Minimum limit | | Maximum limit | |
| --- | --- | --- | --- | --- |
|  | Residues | Atoms | Residues | Atoms |
| 2 | 32 | 202 | 456 | 3288 |
| 3 | 40 | 571 | 1132 | 9188 |
| 4 | 63 | 1013 | 1284 | 9884 |
| 5 | 164 | 2700 | 736 | 11420 |
